# Jie Du Tong Ye San Prevents N-Nitrosomethylbenzylamine-Induced Esophageal Carcinogenesis via Inhibition of Inflammation and Proliferation

**DOI:** 10.1155/2019/5752670

**Published:** 2019-05-20

**Authors:** Simin Zhao, Yanan Jiang, Tongde Tian, Jimin Zhao, Yifei Xie, Xinhuan Chen, Jing Lu, Feng Yang, Honglin Li, Kangdong Liu, Ziming Dong

**Affiliations:** ^1^Department of Pathophysiology, School of Basic Medical Sciences, Zhengzhou University, Zhengzhou 450001, China; ^2^Henan Provincial Cooperative Innovation Center for Cancer Chemoprevention, Zhengzhou University, Zhengzhou 450001, China; ^3^Affiliated Cancer Hospital, Zhengzhou University, Zhengzhou, Henan, China; ^4^School of Pharmacy, East China University of Science and Technology, Shanghai 200237, China

## Abstract

Jie du tong ye san (JDTYS), a traditional Chinese herbal formula, has been used for cancer adjuvant therapy in clinical use and has been shown to be effective in cancer patients. However, the mechanism of JDTYS is still unclear. Therefore, the aim of the present study is to investigate the chemopreventive effects of JDTYS for esophageal squamous cell carcinoma (ESCC) and to clarify the potential mechanism. N-nitrosomethylbenzylamine (NMBA)-induced rat esophageal carcinogenesis was used to evaluate the effect of JDTYS* in vivo*. Rats were treated with NMBA 3 times per week, for a total of 5 weeks. Rats in the treated groups were given JDTYS for 35 weeks. When rats were euthanized, esophageal tissue and blood were collected to evaluate the effects of JDTYS. The pathological grading of the rat esophageal preneoplastic lesions was classified and statistically analyzed. The protein levels of c-Jun and Ki67 were determined by immunohistochemistry. In addition, inflammation markers nuclear factor kappa B (NF-*κ*B), cyclooxygenase-2 (COX-2), and the cluster of differentiation molecule 11B (CD11B) were also determined by immunohistochemistry. Moreover, the expression of COX-2 and Pentraxin 3 (PTX3) in rat serum was determined by enzyme-linked immunosorbent assay (ELISA). JDTYS could inhibit the formation of NMBA-induced esophageal preneoplastic lesions. JDTYS could downregulate the expression of proliferation related proteins Ki67 and c-Jun. Moreover, inflammation related proteins NF-*κ*B, COX-2, and CD11B were inhibited and PTX3 was increased by JDTYS. In all, JDTYS is a promising chemopreventive formula against esophageal carcinogenesis by regulating inflammation and inhibiting cell proliferation.

## 1. Introduction

Esophageal cancer is one of the most lethal cancers and is the leading cause of cancer related death, accounting for more than 450,000 new cases world-wide annually [1, 2]. ESCC, which accounts for over 90% of esophageal cancer, has a complicated etiology [3–6]. Investigations indicate smoking and drinking are the main causes of ESCC in the western world [7, 8]. However, salty food consumption, lack of vitamins and minerals in food, and hot meals and beverages are associated with ESCC in the Far East [8, 9]. Moreover, nitrosamines including NMBA, a potent esophageal carcinogen in human and animals, are also thought to contribute to ESCC burden [10, 11]. The nitrosamines and their precursors are found in the water and food in Linxian, China, which may be responsible for the high incidence of esophageal cancer there. Nitrosamine metabolism causes the methylation of proteins, resulting in gene mutation and carcinogenesis [12, 13]. As well studied, esophageal cancer has high relationship with inflammation [14]. These ESCC risk factors can induce the esophageal epithelium chronic irritation and lead to the occurrence of chronic inflammation. The chronic inflammation can trigger the initiation and progression of dysplasia of esophageal epithelium and finally lead to esophageal cancer [15].

Chinese medicine is getting more attention in chemoprevention research. JDTYS is a Chinese herbal formula which has been clinically used in the treatment of esophagitis and cancer adjuvant therapy. It is made from 11 Chinese crude drugs, including Pu Gong Ying (*Taraxacum mongolicum* Hand.-Mazz.), Gui Zhen Cao (*Bidens bipinnata *Linn.), Teng Li Gen [*Actinidia arguta *(Sieb.et Zucc.) Flarich.ex Miq.], She Gan [*Belamcanda chinensis *(L.) DC.], Zhong Jie Feng [*Sarcandra glabra *(Thunb.) Nakai], Ma Bo (*Lasiosphaera fenzlii *Reich.), Jiang Hou Pu (*Magnolia officinalis *Rehd. Et Wils.), Xi Xian Cao (*Siegesbeckia orientalis *L.), Chan Tui (*Cryptotympana pustulata* Fabr), Jie Geng [*Platycodon grandiflorum *(Jacq.) A.DC.], and Gan Cao (*Glycyrrhiza uralensis* Fisch.). The ratio of the herb is 10: 10: 10: 4: 5: 5: 5: 5: 4: 4: 4. The main components of this formula have anti-inflammation and antitumor effects.* Taraxacum mongolicum* Hand.-Mazz showed effect against inflammation; it may depend on the anti-inflammatory activity of major ingredient organic acid component [16]. The extract from* Actinidia arguta *(Sieb.et Zucc.) Flarich.ex Miq. had an inhibitory effect on hepatocellular carcinoma by inhibiting HCC cell invasion and metastasis [17].* Belamcanda chinensis *(L.) DC also showed antitumor activities [18]. Thus, it is meaningful to assess the effectiveness of JDTYS as a chemopreventive agent for esophageal carcinogenesis.

In the present study, we found that JDTYS can inhibit the formation of preneoplastic lesions induced by NMBA. JDTYS inhibited the expression of cell proliferation related proteins c-Jun and Ki67 and inflammation related proteins NF-*κ*B, COX-2, and CD11B in rat esophageal tissue. JDTYS also inhibited COX-2 expression and increased PTX3 expression in rat serum. Therefore, the inhibitory effect of JDTYS on cell proliferation and inflammation plays an important role in mediating protection against esophageal preneoplastic lesions.

## 2. Materials and Methods

### 2.1. Chemicals Reagents

NMBA was obtained from East China University of Science and Technology with a purity of 98% by high-performance liquid chromatography (Shanghai, China). JDTYS was a gift from Affiliated Cancer Hospital of Zhengzhou University (Henan, China). The antibody to NF-*κ*B p65 was purchased from Santa Cruz Biotechnology (Santa Cruz, CA, USA). The antibodies to c-Jun and COX-2 were purchased from Cell Signaling Biotechnology (Beverly, MA, USA). The antibody to Ki-67 was purchased from Thermo Scientific (Fremont, CA, USA). The antibody to CD11B was obtained from Abcam (Cambridge, UK).

### 2.2. Animals and Diet

The Fisher 344 (F344) rats were purchased from Beijing Vital River (Male, 4-5 weeks old; Beijing, China). 5 rats per cage were group-housed at standard conditions (20 ± 2°C; 50 ± 10% relative humidity; 12 h light/dark cycles). Rats were given the synthetic diet and water* ad libitum* throughout the study. Cages were changed and animal rooms were cleaned every two weeks.

### 2.3. Chemoprevention Bioassay

The F344 rats acclimatized at the new environment for one week after arrival. The rats were randomly assigned to 6 groups: gavaged with water (control group, n = 14); gavaged with 25 g/kg JDTYS (JDTYS control group, n = 4); subcutaneous injection with NMBA 0.5 mg/kg [19] (NMBA group, n = 26); gavaged with 4 g /kg Zengshengping (ZSP) + NMBA 0.5 mg/kg (ZSP group, positive control, n = 6); gavaged with 10 g/kg JDTYS + NMBA 0.5 mg/kg (JDTYS 10 g/kg group, n = 15); and gavaged with 25 g/kg JDTYS + NMBA 0.5 mg/kg (JDTYS 25 g/kg, n = 15). To observe the whole process of esophageal carcinogenesis, rats were sacrificed at different time points. Firstly, 3 rats from the control group and 8 rats from NMBA group were sacrificed at week 15; at week 25, the same number of rats from the control group and NMBA group was sacrificed. The esophagus of the rat was opened longitudinally, kept flat, and the epithelium was exposed on a piece of filter paper and divided into three sections. Half the esophagus was preserved in liquid nitrogen; the other half was fixed for next histopathologic evaluation. At week 35, we euthanized all the rats following the above protocol. We strictly followed the ethical guidelines of institutional, national, or international bodies. The Research Ethics Committee of Zhengzhou University has authorized all the research protocols we submitted.

### 2.4. Histologic Analysis

**Figure 1:**
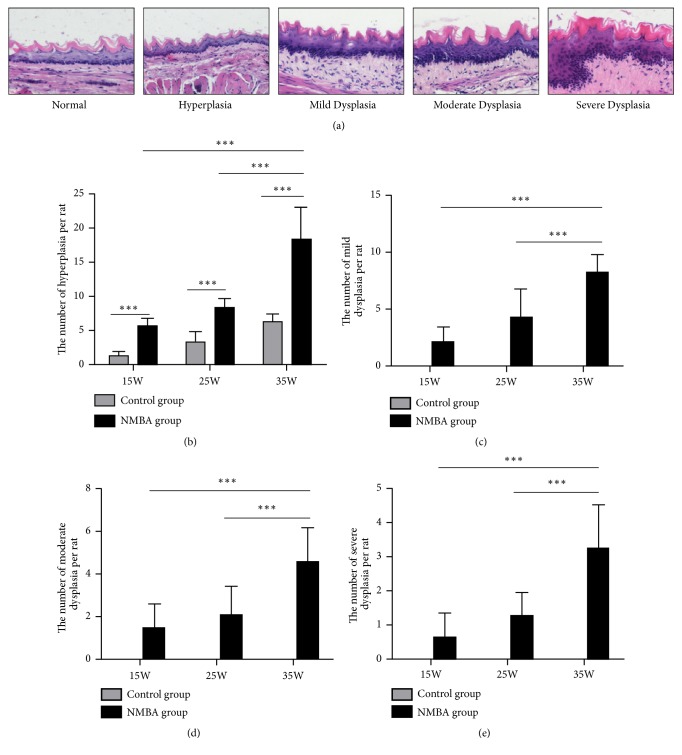
*NMBA induces preneoplastic lesions of rat esophagus.* (a) Pathological changes including normal epithelium, hyperplasia, mild dysplasia, moderate dysplasia, and severe dysplasia in rat esophageal mucosa (200×). (b) The occurrences of hyperplasia had statistical difference in control group and NMBA group at weeks 15, 25, and 35. The NMBA group also showed increased occurrence of hyperplasia at week 35 compared with weeks 15, 25. (c) The incidences of mild dysplasia in NMBA group at week 35 were statistically significant relative to weeks 15, 25. (d) and (e) The occurrences of moderate dysplasia and severe dysplasia in NMBA group at week 35 were higher than at weeks 15, 25 (*∗ P*<0.05, *∗∗ P*<0.01, *∗∗∗ P*<0.001).

The esophagus from each rat was opened longitudinally; then, half of each esophagus was cut into upper, middle, and lower parts. The esophageal tissues were fixed in 10% neutral buffered formalin. All the parts were embedded in paraffin and cut into 4 *μ*m sections, then stained using hematoxylin and eosin (H&E). The grading standard of the rat esophageal tissue was classified according to Gray D. Stoner classification criteria [19]. There are 5 histological categories: normal epithelium, hyperplasia, mild dysplasia, moderate dysplasia, and severe dysplasia. Normal esophageal epithelium usually has normal cell thickness and an orderly basal layer. A little thickening of the basal cell and keratin layers are found in hyperplasia. Obvious thickening of the basal cell and keratin layers are found in moderate dysplasia. Not only more obvious thickening of the keratin layer, but also cellular atypia and disorderly epidermal cells are found in severe dysplasia (Figure 1(a)). Each viewing field under microscope was categorized into different histological categories (normal epithelium, hyperplasia, mild dysplasia, moderate dysplasia, and severe dysplasia). The lesions from three parts of each rat esophagus were counted and the total number of each histological category was recorded.

### 2.5. Immunohistochemistry

The rat esophagus was embedded into paraffin and cut at 4 *μ*m thickness for immunohistochemistry. Slides went through xylene and graded alcohols for deparaffinizing and hydrating. Antigen retrieval was completed using microwave in 10 mM citrate buffer (pH6.0) about 10 min. Ki67 (1:50), c-Jun (1:50), NF-*κ*B (1:100), COX-2 (1:100), CD11B (1:100) were incubated overnight at 4°C. HRP-IgG secondary antibodies were incubated with tissues at 37°C for 15 min. Then the slides were detected with DAB and counterstained by using hematoxylin. Then samples were observed by an Olympus microscope (Tokyo, Japan).

### 2.6. Measurement of COX-2, PTX3 in Plasma

For protein detection, blood was collected from the abdominal aorta and kept 1 h at room temperature, then centrifuged at 3000×g. 100 *μ*l serum from each rat was used to detect COX-2 or PTX3 concentration by using ELISA assay (Cusabio, Houston, TX, USA; Cloud-Clone Corp, Houston, TX, USA).

### 2.7. Statistical Analysis

All quantitative data are expressed as means ± S.E. or S.D. as indicated. A one-way ANOVA was used for statistical analysis. A probability of* p* < 0.05 was used as the criterion for statistical significance.

## 3. Results

### 3.1. The Formation of Rat Esophageal Precancerous Lesions

Histopathological results clearly showed NMBA-induced preneoplastic lesions in the rat esophagus (Figure 1(a)). The NMBA-induced group had increased occurrences of hyperplasia compared with the control group at weeks 15, 25, and 35; the occurrence of hyperplasia at week 35 was statistically different from weeks 15, 25 (Figure 1(b)); the NMBA-induced group also had significant increased occurrences of mild dysplasia, moderate dysplasia, and severe dysplasia at week 35 compared with weeks 15, 25 (Figures 1(c), 1(d) and 1(e)).

### 3.2. Effects of JDTYS on NMBA-Induced Preneoplastic Lesions

**Figure 2:**
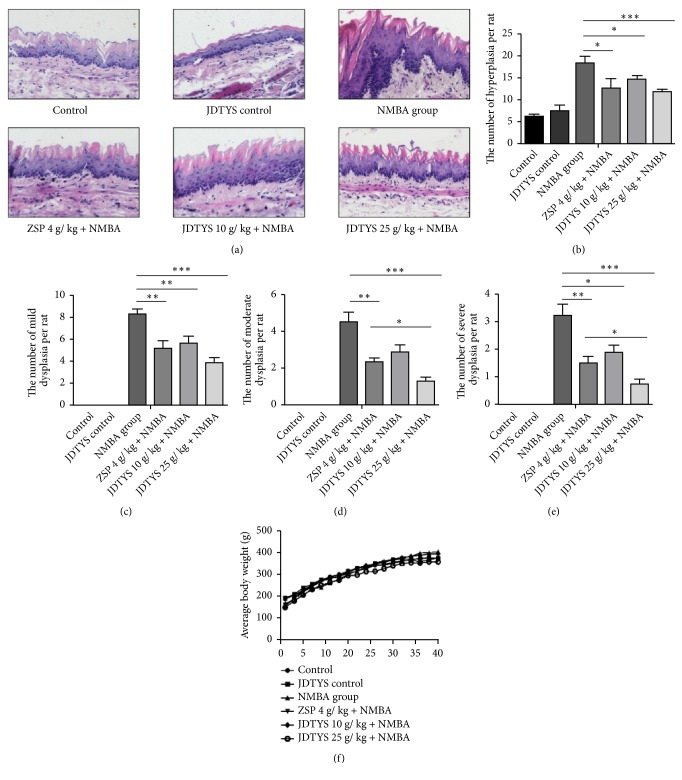
*JDTYS inhibits NMBA-induced preneoplastic lesions.* (a) Representative figure of each group stained with H&E (200×). (b) At week 35, the occurrences of esophageal mucosal hyperplasia, in the ZSP, JDTYS 10 g/kg, and JDTYS 25 g/kg groups compared with the NMBA group. (*∗ P*<0.05, *∗∗ P*<0.01, *∗∗∗ P*<0.001). (c) At week 35, the mild dysplasia in the ZSP, JDTYS 10 g/kg, and JDTYS 25 g/kg groups was statistically different compared with the NMBA group. (d) The ZSP, JDTYS 10 g/kg and JDTYS 25 g/kg groups all significantly reduced the occurrences of moderate dysplasia (*∗ P*<0.05, *∗∗ P*<0.01, *∗∗∗ P*<0.001). (e) At week 35, the severe dysplasia in the ZSP, JDTYS 10 g/kg, and JDTYS 25 g/kg groups was statistically different compared with the NMBA group (*∗ P*<0.05, *∗∗ P*<0.01, *∗∗∗ P*<0.001). (f) JDTYS has no effect on the body weight of rats.

The pathological changes of rat esophageal mucosa in different groups are demonstrated in Figure 2(a). At week 35, the occurrences of hyperplasia were reduced in the JDTYS 10 g/kg group, 25 g/kg, and ZSP groups when compared with the NMBA group (Figure 2(b)); the occurrences of rat esophageal mild dysplasia were inhibited in the JDTYS 10 g/kg, JDTYS 25 g/kg, and ZSP groups (Figure 2(c)); there were also significant differences in the occurrences of moderate dysplasia and severe dysplasia in the 3 groups relative to the NMBA group (Figures 2(d) and 2(e)). Our results indicated that both ZSP and JDTYS can inhibit precancerous lesions induced by NMBA; furthermore, the inhibition effect of JDTYS 25 g/kg on moderate and severe dysplasia is stronger than that of the ZSP group.

### 3.3. General Observations

There were no significant differences between the experimental group and the control group in rat average body weights (Figure 2(f)). There were also no differences in drink and food consumption.

### 3.4. Effects of JDTYS on the Expression of Ki67 and c-Jun

**Figure 3:**
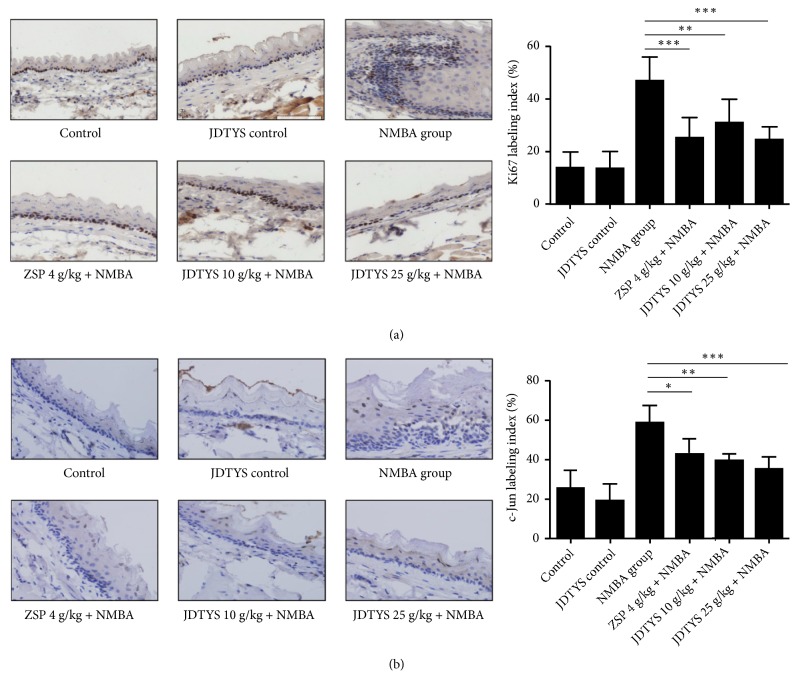
*Effects of JDTYS on expression of proliferation markers in the rat at week 35.* (a) Immunohistochemistry analysis was used to determine the level of Ki67 in the ZSP, JDTYS 10 g/kg, and JDTYS 25 g/kg groups compared with the NMBA group; there were significant differences (200×; *∗P*<0.05, *∗∗P*<0.01, *∗∗∗P*<0.001). (b) Immunohistochemistry analysis for c-Jun: the expression levels of c-Jun were significantly inhibited in the ZSP, JDTYS 10 g/kg, and JDTYS 25 g/kg groups compared with the NMBA group (200×; *∗P*<0.05, *∗∗ P*<0.01, *∗∗∗ P*<0.001).

The expression of Ki67 and c-Jun were observed by immunohistochemistry analysis. The Ki67 protein expressed in the nucleus was significantly upregulated in the NMBA-induced rat esophageal mucosa compared with the control group. The JDTYS 10 g/kg, JDTYS 25 g/kg, and ZSP groups all significantly reduced the expression of Ki67 protein compared with the NMBA group (Figure 3(a)). Immunohistochemistry result of c-Jun also showed nuclear staining and was mainly localized in the suprabasal layer of the esophageal epithelium. The expression of c-Jun was reduced in the JDTYS 10 g/kg, JDTYS 25 g/kg, and ZSP groups compared with the NMBA group (Figure 3(b)).

### 3.5. Effects of JDTYS on the Inflammation Related Protein

**Figure 4:**
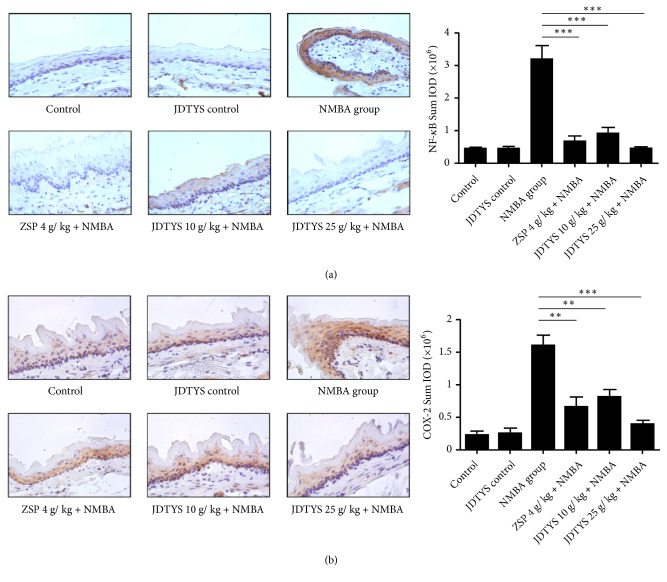
*Effects of JDTYS on expression of inflammatory factors in the rat at week 35.* (a) Immunohistochemistry analysis for NF-*κ*B. The ZSP, JDTYS 10 g/kg, and JDTYS 25 g/kg groups showed reduced expression of NF-*κ*B compared with the NMBA group (200×; *∗P*<0.05, *∗∗ P*<0.01, *∗∗∗ P*<0.001). (b) Rats esophageal epithelium in each group was stained with COX-2. The ZSP, JDTYS 10 g/kg, and JDTYS 25 g/kg groups significantly inhibited the expression of inflammation related protein COX-2 (200×; *∗P*<0.05, *∗∗ P*<0.01, *∗∗∗ P*<0.001).

**Figure 5:**
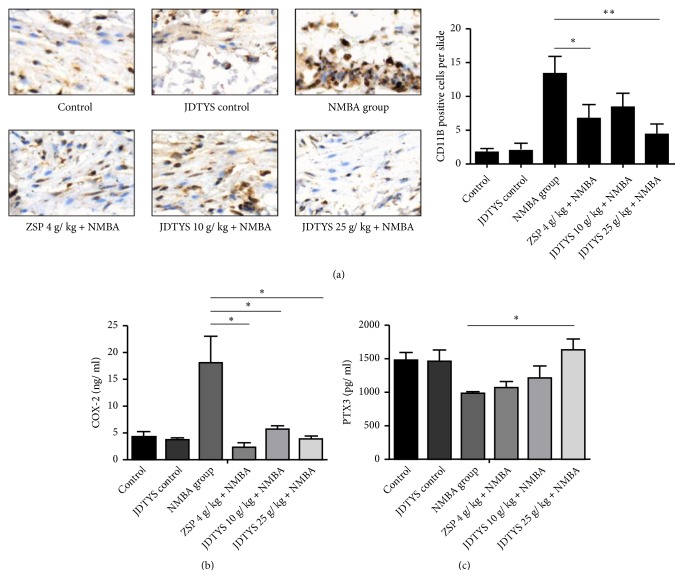
*Effects of JDTYS on expression of CD11B in the rat esophagus and COX-2, PTX3 expression in rat serum via immunoblotting and ELISA.* (a) Rats esophageal epithelium samples were harvested and then stained with CD11B antibody. Expression of CD11B was calculated using positive staining cells. Each slide was counted, 5 separate areas. The JDTYS 10 g/kg and JDTYS 25 g/kg groups significantly reduced the expression of CD11B (400×; *∗P*<0.05, *∗∗ P*<0.01, *∗∗∗ P*<0.001). (b) Collect rat serum to detect COX-2 expression. The ZSP, JDTYS 10 g/kg, and JDTYS 25 g/kg groups significantly reduced the expression of COX-2 in rat serum (*∗P*<0.05). (c) Rat serum was used to detect PTX3 expression. The JDTYS 25 g/kg group significantly increased the expression of PTX3 in rat serum (*∗P*<0.05).

Inflammation contributes to the carcinogenesis of esophageal cancer [15, 20]. Thus, we checked whether JDTYS can modulate the inflammation level after NMBA being induced. In many cancers NF-*κ*B is activated and plays a role in protumorigenic functions [21]. NF-*κ*B p65 was significantly inhibited in the JDTYS 10 g/kg, JDTYS 25 g/kg, and ZSP groups compared with the NMBA group (Figure 4(a)). COX-2 can catalyze the synthesis of prostaglandins and function as a proinflammatory factor [22]. The JDTYS 10 g/kg, JDTYS 25 g/kg, and ZSP groups all significantly inhibited the expression of COX-2 (Figure 4(b)). In the JDTYS 10 g/kg group and the JDTYS 25 g/kg group, CD11B staining cells were also reduced compared with the NMBA group (Figure 5(a)). At 35w, COX-2 level was inhibited in rat serum of the ZSP, JDTYS 10 g/kg, and JDTYS 25 g/kg groups compared with the NMBA group. So, JDTYS significantly reduced COX-2 production in rat serum treated with JDTYS (Figure 5(b)). PTX3 deficiency triggers complement-dependent tumor-promoting inflammation. We found, at week 35, the JDTYS 25 g/kg group had higher serum PTX3 level when compared with the NMBA group (Figure 5(c)). Collectively, these results suggest that JDTYS inhibited NMBA-induced preneoplastic lesions by reducing inflammation.

## 4. Discussion

The development of ESCC undergoes a long process from initiation to progression. It has many stages including hyperplasia, mild dysplasia, moderate dysplasia, severe dysplasia, carcinoma* in situ,* and ESCC. More importantly, the precancerous lesions of esophageal cancer have two-way instability characteristics, with the possibility of developing to cancer or reversing this development by early intervention. Therefore, this characteristic provides an opportunity to intervene ESCC. Chemoprevention has been regarded as a promising way to prevent ESCC. It had been reported that black raspberries or their polyphenolic anthocyanins inhibit esophageal tumorigenesis by their inhibitory effects on genes associated with inflammation [23]. However, their effectiveness in the human population still needs further investigation. Thus, it is still urgent to find a promising and safe drug against esophageal cancer. In this study, we confirmed that JDTYS significantly inhibited esophageal preneoplastic lesions formation in rat treated with NMBA. Importantly, the inhibitory function of JDTYS high dose on moderate dysplasia and severe dysplasia of rat esophageal epithelium had significant difference compared with ZSP, which is traditional Chinese medicine for esophageal preneoplastic lesions treatment [24, 25].

C-Jun is a positive regulator of cell proliferation and is activated by JNK, to promote the cell cycle [26]. JDTYS significantly inhibited the expression of c-Jun. Ki67, a nuclear antigen that indicates the status of cell proliferation, was strongly inhibited after JDTYS treatment. Chronic, dysregulated, persistent, and unresolved inflammation all can lead to an increased risk of malignant disease [27]. In the process of esophageal cancer development, cancer related inflammation has an important role [28]. The nuclear factor NF-*κ*B pathway as a proinflammatory signaling pathway is involved in many human cancers. NF-*κ*B as a transcriptional regulator can increase the expression of the* cox-2* gene [29, 30]. COX-2 also has an important function in pathological processes such as cancer initiation [31–33]. JDTYS significantly inhibited the expression of NF-*κ*B p65 in rat esophageal epithelium. JDTYS also significantly decreased the expression of COX-2 in rat esophageal epithelium and serum. In addition, PTX3 as an essential component of the humoral arm of innate immunity plays an important role in the regulation of inflammation [34, 35]. It also has been reported that PTX3 attenuates inflammation through regulation of macrophage activity [36]. Our results showed JDTYS upregulated the expression of PTX3 and inhibited the expression of CD11B serving as a marker of macrophages.

In summary, we provided evidence that JDTYS significantly inhibits the formation of esophageal precancerous lesions induced by NMBA. The inhibitory effect of JDTYS on esophageal precancerous lesions is related to downregulation of proliferation related proteins Ki67 and c-Jun and modulation inflammation related proteins COX-2, NF-*κ*B, PTX3, and CD11B.

## 5. Conclusion

Collectively, our data suggest that JDTYS can prevent esophageal carcinogenesis by inhibiting cell proliferation and downregulating inflammation. So JDTYS may be a promising chemoprevention drug for esophageal carcinogenesis.

## Data Availability

The data used to support the findings of this study are available from the corresponding author upon request.

## Abbreviations

JDTYS: Jie du tong ye san
ESCC: Esophageal squamous cell carcinoma
NMBA: N-nitrosomethylbenzylamine
ZSP: Zengshengping
NF-*κ*B: Nuclear factor kappa B
COX-2: Cyclooxygenase-2
PTX3: Pentraxin 3
CD11B: The cluster of differentiation molecule 11B
ELISA: Enzyme-linked immunosorbent assay
H&E: Hematoxylin and eosin.
